# Empagliflozin improves cardiac energetics during ischaemia/reperfusion by directly increasing cardiac ketone utilization

**DOI:** 10.1093/cvr/cvad157

**Published:** 2023-10-11

**Authors:** Dylan Chase, Thomas R Eykyn, Michael J Shattock, Yu Jin Chung

**Affiliations:** British Heart Foundation Centre of Research Excellence, King’s College London, The Rayne Institute, 4th Floor, Lambeth Wing, St Thomas’ Hospital, London SE1 7EH, UK; British Heart Foundation Centre of Research Excellence, King’s College London, The Rayne Institute, 4th Floor, Lambeth Wing, St Thomas’ Hospital, London SE1 7EH, UK; School of Biomedical Engineering and Imaging Sciences, King’s College London, London SE1 7EH, UK; British Heart Foundation Centre of Research Excellence, King’s College London, The Rayne Institute, 4th Floor, Lambeth Wing, St Thomas’ Hospital, London SE1 7EH, UK; British Heart Foundation Centre of Research Excellence, King’s College London, The Rayne Institute, 4th Floor, Lambeth Wing, St Thomas’ Hospital, London SE1 7EH, UK

**Keywords:** SGLT2 inhibitors, Empagliflozin, Sodium-glucose transporter 2, Ischaemia-reperfusion, Metabolism, Substrate utilisation, Ketones, Isolated heart, Heart failure

## Abstract

**Aims:**

Empagliflozin (EMPA), a potent inhibitor of the renal sodium–glucose cotransporter 2 and an effective treatment for Type 2 diabetes, has been shown to have cardioprotective effects, independent of improved glycaemic control. Several non-canonical mechanisms have been proposed to explain these cardiac effects, including increasing circulating ketone supply to the heart. This study aims to test whether EMPA directly alters cardiac ketone metabolism independent of supply.

**Methods and results:**

The direct effects of EMPA on cardiac function and metabolomics were investigated in Langendorff rat heart perfused with buffer containing 5 mM glucose, 4 mM β-hydroxybutyrate (βHb) and 0.4 mM intralipid, subject to low flow ischaemia/reperfusion. Cardiac energetics were monitored *in situ* using ^31^P NMR spectroscopy. Steady-state ^13^C labelling was performed by switching ^12^C substrates for ^13^C_1_ glucose or ^13^C_4_ βHb and ^13^C incorporation into metabolites determined using 2D ^1^H-^13^C HSQC NMR spectroscopy. EMPA treatment improved left ventricular-developed pressure during ischaemia and reperfusion compared to vehicle-treated hearts. In EMPA-treated hearts, total adenosine triphosphate (ATP) and phosphocreatine (PCr) levels, and Gibbs free energy for ATP hydrolysis were significantly higher during ischaemia and reperfusion. EMPA treatment did not alter the incorporation of ^13^C from glucose into glycolytic products lactate or alanine neither during ischaemia nor reperfusion. In ischaemia, EMPA led to a decrease in ^13^C_1_ glucose incorporation and a concurrent increase in ^13^C_4_ βHb incorporation into tricarboxylic acid (TCA) cycle intermediates succinate, citrate, and glutamate. During reperfusion, the concentration of metabolites originating from ^13^C_1_ glucose was similar to vehicle but those originating from ^13^C_4_ βHb remained elevated in EMPA-treated hearts.

**Conclusion:**

Our findings indicate that EMPA causes a switch in metabolism away from glucose oxidation towards increased ketone utilization in the rat heart, thereby improving function and energetics both during ischaemia and recovery during reperfusion. This preference of ketone utilization over glucose was observed under conditions of constant supply of substrate, suggesting that EMPA acts directly by modulating cardiac substrate preference, independent of substrate availability. The mechanisms underlying our findings are currently unknown, warranting further study.

## Graphical Abstract

**Graphical Abstract:**
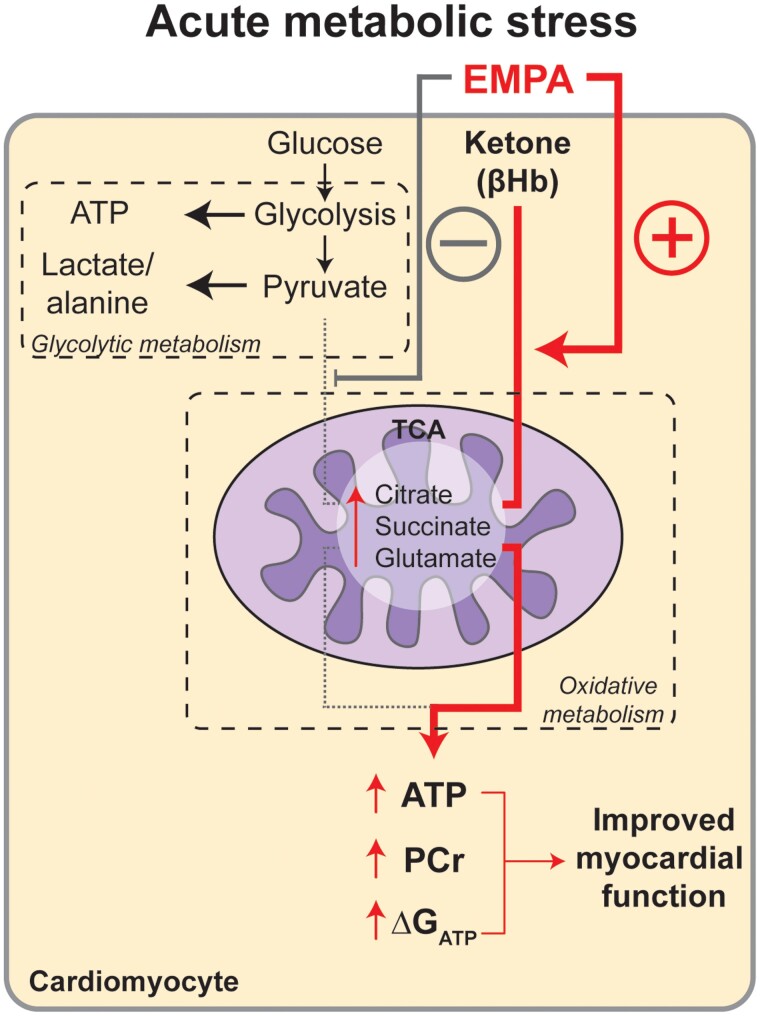

**Time of primary review: 19 days**

## 1. Introduction

Empagliflozin (EMPA), an inhibitor of renal sodium–glucose cotransporter 2 (SGLT2), is used clinically in the treatment of Type 2 diabetes.^1^ The EMPA-REG OUTCOME trial, however, first described an unexpected beneficial cardiovascular effect associated with EMPA, notably a reduction in heart failure (HF)-related hospitalization independent of improved glycaemic control.^2^ More recently, the EMMY trial has also demonstrated cardioprotective benefits of EMPA in patients with recent myocardial infarction, where patients receiving EMPA show significant improvement in cardiac function and structural parameters compared to the placebo cohort.^3^ These clinical observations are supported by preclinical studies in animal models, with or without diabetes, showing that HF and pathological cardiac remodelling are alleviated by EMPA.^4–6^ These cardioprotective effects are observed despite cardiomyocytes not expressing SGLT2,^7–9^ leading to suggestion that SGLT2 inhibitors (SGLT2i's) may modulate other aspects of cardiac physiology.

In HF, abnormalities in myocardial energetics precede and contribute to the disease progression.^10–12^ Depending on substrate availability, the heart possesses the flexibility to utilize a wide range of substrates to meet metabolic demand, such as free fatty acids, glucose, and ketone bodies. Mounting evidence suggests a possible metabolic effect of SGLTi's. A recent study in db/db mice showed that EMPA treatment leads to significantly increased cytosolic and mitochondrial adenosine triphosphate (ATP) levels as well as improved maintenance of ATP both during ischaemia/reperfusion (I/R) in isolated hearts and during hypoxia in isolated cardiomyocytes.^13^ This study suggests EMPA has a direct metabolic effect on energetics when the heart is stressed either by I/R, hypoxia, HF or diabetes.

A ‘thrifty substrate’ hypothesis has been suggested as the mechanism by which SGLT2i's confer cardioprotection, i.e. SGLT2i's alter myocardial metabolism to favour ketone oxidation—an energetically efficient substrate.^14^ This could occur (i) indirectly by increasing circulating ketone levels and/or (ii) directly by altering myocardial substrate preference independent of substrate supply. Indeed, an increase in circulating plasma ketone bodies has been previously reported in EMPA-treated animals^4,15,16^ and in humans.^14,17–19^ This has led to numerous studies reporting that the cardioprotective effects of EMPA are attributable to improved cardiac efficiency via enhanced cardiac ketone utilization, only secondary to increased availability of circulating ketone bodies.^14,19^ However, direct evidence of altered myocardial ketone body utilization in response to EMPA, independent of substrate supply, is lacking.

In this study, we used isolated Langendorff-perfused healthy rat hearts to test whether the SGLT2i EMPA, under conditions of unchanging substrate supply, can induce a switch towards the energetically favourable oxidation of ketones and away from glucose utilization. Hearts were subject to low flow (10%) ischaemia as a metabolic stressor in which oxidative metabolism is compromised, but not eliminated, and anaerobic metabolism is upregulated. We observe an EMPA-induced increase in metabolic efficiency via (i) improved function and recovery as well as improved Gibbs free energy for ATP hydrolysis during both ischaemia and reperfusion, (ii) improved PCr/ATP ratios, (iii) increased ^13^C_4_ βHb oxidation and reduced ^13^C_1_ glucose oxidation during ischaemia, and (iv) increased ^13^C_4_ βHb oxidation during reperfusion. The results of this study provide evidence, for the first time, of a direct cardiac response through which EMPA modulates metabolic substrate preference in a metabolically stressed heart. The mechanism underlying this finding and its relevance to chronic HF are currently unknown and warrant further investigation.

## 2. Methods

### 2.1 Approvals for animal work

Animal procedures were performed in compliance with the guidelines from Directive 2010/63/EU of the European Parliament on the protection of animals used for scientific purposes, Home Office Guidance on the Operation of the Animals (Scientific Procedures) Act of 1986, and the King’s College London institutional guidelines. Male Wistar rats (∼225 g) were purchased through Envigo. Animals were euthanized humanely through anaesthetic overdose via IP injection of pentobarbital sodium pentobarbitone (60 mg/kg) and death confirmed by exsanguination.

### 2.2 Chemicals

EMPA was obtained from Boehringer Ingelheim. ^13^C_1_ D-glucose and ^13^C_4_ sodium DL-3-hydroxybutyrate were purchased from Sigma.

### 2.3 Langendorff perfusion

Hearts were rapidly excised and cannulated via the aorta and perfused at 37°C at constant pressure of 80 mmHg. Perfusion buffer was a modified Krebs–Henseleit buffer (KHB) containing (in mM): 114 NaCl, 5.9 KCl, 1.16 MgSO_4_, 25 NaHCO_3_, 0.48 EDTA, 2.2 CaCl_2_, 1 Na L-lactate, 0.1 Na pyruvate, 0.5 L-glutamic acid, 5mU/L insulin, 0.4 intralipid 20%, 5 glucose, and 4 DL-β-hydroxybutyric acid sodium salt and bubbled with 5% CO_2_/95% O_2_. Intralipid 20% is clinically used as the source of fatty acids^20^ and is composed of: linoleic acid (44–62%), oleic acid (19–30%), palmitic acid (7–14%), α-linolenic acid (4–11%), stearic acid (1.5–5.5%), and egg yolk phosphatide (1.2%). The low flow ischaemia protocol consisted of 20 min baseline, 40 min ischaemia at a flow rate equivalent to 10% of baseline flow, and 30 min reperfusion at a flow rate determined by a constant perfusion pressure of 80 mmHg. ^13^C labelling was achieved by switching to either ^13^C_1_ D-glucose or ^13^C_4_ sodium DL-3-hydroxybutyrate for 10 min. EMPA or DMSO (v/v) was added after 10 min of baseline perfusion and maintained in the perfusion buffer throughout the entire protocol, such that hearts were pre-treated for 10 min prior to ischaemia. In a separate protocol, hearts were perfused in KHB buffer containing the substrates ^13^C_1_ glucose and lipids, but not β-Hb for 20 min baseline followed by 20 min EMPA or DMSO (v/v) treatment to assess the effect of EMPA on cardiac metabolism in the absence of ketone substrate. Cardiac function was monitored throughout via a balloon inserted into the left ventricle and monitored on LabChart (AD Instruments, Australia). The Langendorff rig was modified for nuclear magnetic resonance (NMR) as described previously.^21^

### 2.4 Dual phase metabolite extraction

Snap frozen hearts were pulverized to fine powder over liquid nitrogen. Approximately 0.8–1 g of cardiac tissue was used for the extraction. The tissue was homogenized using a TissueRuptor in 4 mL of ice-cold methanol over ice. Subsequently, the following was added with vortexing between additions: 2 mL ice-cold ddH_2_O, 2 mL ice-cold chloroform, 1.8 mL ice-cold ddH_2_O, and 2 mL ice-cold chloroform. Samples were centrifuged at 3600 *g*, 4°C for 60 min. The aqueous phase was transferred to a fresh tube containing Chelex and centrifuged for an additional 5 min. The supernatant was transferred to pre-weighed fresh tubes containing 15 µL pH indicator and snap frozen in liquid nitrogen until frozen and then freeze-dried at −20°C. A hole was made on the lid of each tube to allow for the sublimation to escape the tubes.

### 2.5 NMR spectroscopy

^31^P spectra were acquired on a Bruker Avance III 400 MHz Spectrometer 9.4 T vertical-bore magnet using a 15 mm dual tuned ^31^P/^1^H coil. Spectra were acquired and analysed using TopSpin 3.7pl software. For every heart, shimming was carried out on the ^1^H channel to yield a water linewidth of <50 Hz. Time-resolved ^31^P spectra were acquired with a 60° flip angle, 64 scans, and a total acquisition time of 4 min for a single spectrum employing a pre-scan delay of 3 s, 16k data points, and an acquisition time of 0.85 s. An exponential line broadening factor of 25 Hz was applied prior to Fourier transformation, phase, and baseline correction.

Peak areas of the Pi, PCr, and β-ATP peaks were measured in TopSpin and normalized to the PCr peak during the baseline stability period. Intracellular pH was calculated according to the equation:


(1) $$\mathrm{pH}=6.694+\mathrm{lo}g_{10}\left(\frac{\Delta \mathrm{ppm}-3.121}{5.498-\Delta \mathrm{ppm}}\right),$$

where Δppm is the chemical shift difference between the PCr and Pi peaks. The Gibbs free energy for ATP hydrolysis ΔG_ATP_ is given by


(2) $$\begin{array}{r} \Delta G_{\mathrm{ATP}}=\Delta G_{0}+\mathrm{RTln}\left(\frac{\left[\mathrm{ADP}][Pi\right]}{\left[\mathrm{ATP}\right]}\right)=\Delta G_{0}+\mathrm{RTln}\left(\frac{\left[\mathrm{Cr}][\mathrm{Pi}\right]}{[\mathrm{PCr}][H^{+}]K_{\mathrm{eq}}}\right), \end{array}\;$$

where the creatine kinase equilibrium constant is given by K_eq_ = 1.66 × 10^9^ mol^−1^ L^−1^ and ΔG_0_ = −30.5 kJ/mol.^22^ Intracellular [Cr] = 20 mM was assumed to be constant during the perfusion.

^13^C labelling was quantified using the same Bruker Avance III 400 MHz Spectrometer 9.4 T vertical-bore magnet equipped with a BBO probe. 2D gradient selected ^1^H/^13^C HSQC (hsqcetgp) experiments were acquired with 16 scans, 1024 data points in the direct dimension, 512 data points in the indirect dimension, and an experiment duration of 3 h 30 min. 2D HSQC data were processed with a QSINE function in both dimensions prior to Fourier transformation, phase, and baseline correction. Peak assignments were performed using the respective assignments from the 1D ^1^H data. Two-dimensional peak integration was performed in TopSpin and normalized relative to the TSP peak.

### 2.6 Statistics

Data are reported as mean ± SEM. Two-tail, unpaired Student’s *t*-test was used to compare EMPA group mean with the control (DMSO) group. *P < 0.05, **P < 0.01, ***P < 0.001, and ****P < 0.0001. Data analysis was carried out in a blinded manner.

## 3. Results

### 3.1 Supply of ketone improves energetics and function in healthy, normoxic hearts

**Figure 1:**
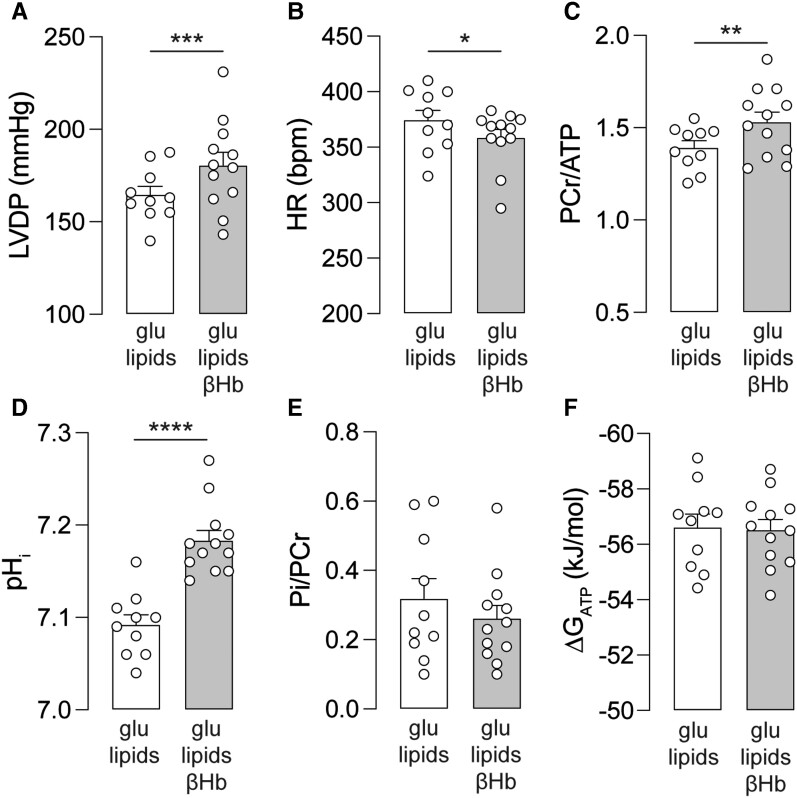
Cardiac function and energetics in the absence or presence of βHb. (*A*) Left ventricular developed pressure, (*B*) heart rate, (*C*) PCr/ATP ratio obtained by ^31^P NMR spectroscopy, (*D*) intracellular pH (pH_i_) obtained by ^31^P NMR spectroscopy, (*E*) inorganic phosphate to PCr ratio obtained by ^31^P NMR spectroscopy, and (*F*) free energy of ATP hydrolysis (ΔG_ATP_) in hearts perfused in either ketone-free (glu + lipids) or ketone-supplemented (glu + lipids + βHb) KH buffer. *n* = 10 rats (glu lipids) or 12 rats (glu+lipids+βHb). *P < 0.05, **P < 0.01, ***P < 0.001, ***P < 0.0001. Data plotted as mean ± SEM.

Under aerobic conditions, the addition of βHb to the perfusate significantly increased left ventricular (LV) function compared to ketone-free conditions (ketone-free: 165 ± 4.6 mmHg vs. ketone-supplemented: 181 ± 7.0 mmHg; *Figure 1A*), whilst reducing heart rate (HR; ketone-free: 374 ± 8.9 bpm vs. ketone-supplemented: 359 ± 7.5 bpm; *Figure 1B*). These functional changes were accompanied by increased PCr/ATP ratio in the presence of ketones (ketone-free: 1.39 ± 0.04 vs. ketone-supplemented: 1.53 ± 0.05; *Figure 1C*). In addition, hearts supplemented with βHb had pH_i_ values of 7.2, which is more consistent with physiological pH_i_, compared to the slightly acidic pH_i_ of 7.1 achieved in hearts perfused without βHb (*Figure 1D*). The Pi/PCr ratio (*Figure 1E*) and Gibbs free energy of ATP hydrolysis (ΔG_ATP_; *Figure 1F*) were unaffected by the presence of ketones in the perfusate. Thus, supplementing the heart with ketones, in addition to glucose and lipids, appears to create a more favourable metabolic condition for the heart whilst not affecting overall energetics.

### 3.2 Supply of ketone reduces glycolysis and glucose oxidation in healthy, normoxic hearts

**Figure 2:**
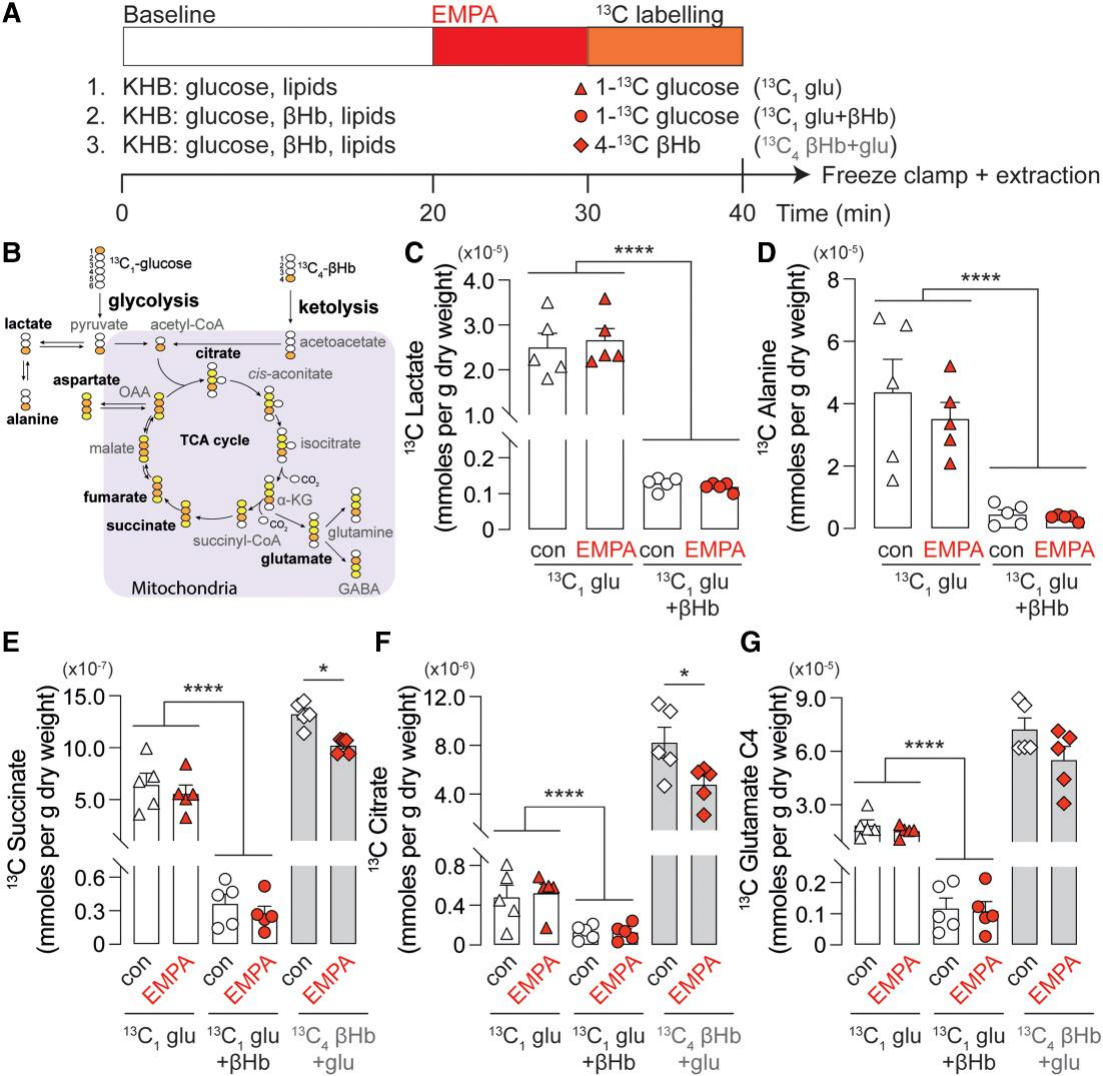
Effect of EMPA on cardiac metabolomics under healthy (baseline) conditions. (*A*) Schematic of perfusion and ^13^C labelling protocol. Hearts were perfused in either a ketone-free (buffer conditions 1, glu) or ketone-supplemented (buffer conditions 2 and 3, glu + βHb) KHB. Hearts were perfused for 20 min to establish baseline and then treated with EMPA or DMSO for 20 min. Hearts were ^13^C labelled for 10 min with either ^13^C-glucose or ^13^C-βHb in the presence of EMPA or DMSO prior to snap freezing in liquid nitrogen. (*B*) Diagram of ^13^C incorporation into metabolic cycle originating from either glucose or β-Hb. (*C*) ^13^C lactate and (*D*) ^13^C alanine originating from ^13^C glucose in hearts perfused in ketone-free (^13^C_1_ glu) or ketone-supplemented (^13^C_1_ glu + βHb) buffer. (*E*) ^13^C succinate (*F*) ^13^C citrate and (*G*) ^13^C glutamate originating from ^13^C glucose in hearts perfused in (i) ketone-free (^13^C_1_ glu) or (ii) ketone-supplemented (^13^C_1_ glu + βHb) buffer or (iii) ^13^C_4_ βHb in hearts perfused in ketone-supplemented buffer (^13^C_4_ βHb + glu). *n* = 5 rats per condition. *P < 0.05, ****P < 0.0001. Data plotted as mean ± SEM.

Given the overall improvement in function in the presence of ketones, we next sought to determine the contribution of ketones to cardiac substrate preference in healthy normoxic hearts perfused with KHB containing lipids and glucose, with (^13^C_1_ glu + ^12^C βHb or ^13^C_4_ βHb + ^12^C glu) or without βHb (^13^C_1_ glu) (*Figure 2*, *compare bars with white symbols*). The metabolomic profile was obtained by labelling the metabolites arising from either ^13^C_1_ glucose or ^13^C_4_ βHb and the extracted cardiac metabolites analysed using ^1^H-^13^C 2D HSQC NMR spectroscopy (see Supplementary material online, *Figure S1*).

When supplied with unlabelled βHb, the concentration of glycolytic products lactate (*Figure 2C*) and alanine derived from ^13^C_1_ glucose (*Figure 2D*) was significantly reduced when compared to conditions lacking ketone supply. Similarly, ^13^C_1_ glucose oxidation through the tricarboxylic acid (TCA) cycle was significantly reduced in the presence of unlabelled ketones as evidenced by the reduction in the concentrations of ^13^C succinate (*Figure 2E*), citrate (*Figure 2F*), and glutamate (*Figure 2G*). Concurrently, the concentration of these metabolites originating from ^13^C_4_ βHb was significantly higher compared to those produced from ^13^C_1_ glucose. Taken together, these results demonstrate a shift in cardiac substrate utilization away from glucose metabolism, both glycolytic and oxidative, towards ketone oxidation in the presence of βHb in the perfusate under normoxic conditions.

### 3.3 EMPA reduces ketone utilization in healthy, normoxic hearts

In aerobically perfused hearts, EMPA treatment (20 min) did not affect ^13^C_1_ glucose utilization, either in the presence or in the absence of ketone supply (*Figure 2E–G*, triangle and circle data points). Interestingly, the drug appeared to reduce ^13^C_4_ ketone oxidation (diamond data points)—notably, concentrations of succinate and citrate derived from βHb were significantly reduced in EMPA-treated hearts compared to controls.

### 3.4 EMPA improves cardiac function during ischaemia and reperfusion

To date, the cardioprotective effects of EMPA have been observed under pathological conditions, such as myocardial infarction (M/I) or HF.^4,5,15^ To mimic a pathological state for healthy hearts in the acute setting, hearts were metabolically stressed by subjecting to low flow ischaemia for 40 min, where perfusion was reduced to 10% of the baseline flow rate. Hearts were then reperfused for 30 min. Hearts were supplied with glucose, ketones, and lipids as available energetic substrates and treated with EMPA or DMSO.

**Figure 3:**
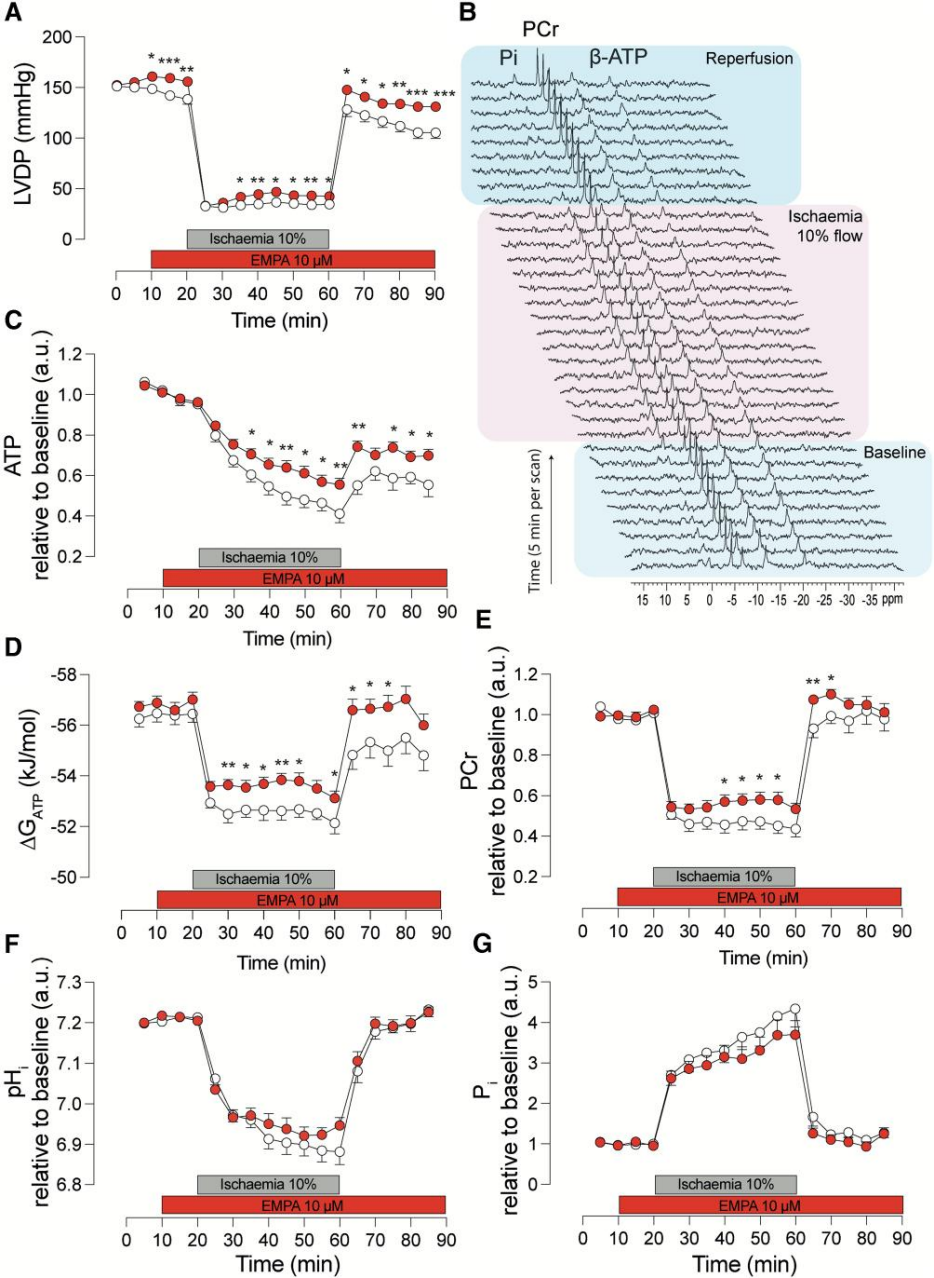
EMPA improves cardiac function and energetics in ischaemia/reperfusion. (*A*) Time course of left ventricular developed pressure measured before (baseline), during and after ischaemia and reperfusion. Hearts were treated with EMPA or DMSO after obtaining 10 min of stable baseline and 10 min prior to ischaemia. (*B*) Example of serially obtained ^31^P NMR spectra. Time course of (*C*) ATP, (*D*) Gibbs free energy of ATP hydrolysis (ΔG_ATP_), (*E*) PCr, (*F*) intracellular pH, and (*G*) inorganic phosphate during ischaemia/reperfusion. *n* = 30 rats (up to ischaemia) and *n* = 16 rats (up to reperfusion) per condition. *P < 0.05, **P < 0.01, and ***P < 0.001. Data plotted as mean ± SEM.

Treatment with 10 µM EMPA improved baseline cardiac function by approximately 8–12% over the course of 10 min compared to control hearts (*Figure 3A*). This improvement was not observed when hearts were supplied only with glucose and lipids and not ketones (previously published data, Chung *et al*.^23^) suggesting ketones may be the substrate through which EMPA acts to modulate cardiac function.

During ischaemia in both EMPA-treated and control hearts, LV function rapidly reduced to ∼30% of baseline (*Figure 3A*). However, after 15 min of ischaemia, LV function of EMPA-treated hearts was significantly higher than that in control hearts [LV developed pressure (LVDP) range 42–47 mmHg vs. 34–36 mmHg, EMPA vs. control, respectively] with ischaemic contractility maintained approximately 25% above that seen in control hearts throughout the last 25 min of ischaemia. On reperfusion, functional recovery was also significantly improved in EMPA-treated hearts, with LVDP 15% higher than controls. Furthermore, during the 30 min of reperfusion, whereas the function of control hearts gradually reduced by 18% (128 ± 2.7 mmHg at 65 min vs. 105 ± 5.7 mmHg at 90 min), the function of EMPA-treated hearts was only reduced by 9% (148 ± 2.7 mmHg at 65 min vs. 131 ± 3.2 mmHg at 90 min).

### 3.5 EMPA improves ATP, PCr, and Gibbs free energy of ATP hydrolysis during ischaemia and reperfusion

The functional improvement observed in EMPA-treated hearts during ischaemia and reperfusion may be due to altered energetics in these hearts. We therefore investigated the energetics of I/R hearts using serially acquired ^31^P NMR spectroscopy in the presence or absence of EMPA (*Figure 3B*). As expected, ATP and PCr levels, as well as Gibbs free energy and pH_i_, were reduced, whilst Pi was elevated during ischaemia in both EMPA and control hearts (*Figure 3C–G*); these changes were subsequently reversed towards their respective baseline levels during reperfusion. However, EMPA significantly preserved the level of these metabolites and the free energy during ischaemia, with ATP and PCr 35%, 23% greater, and Gibbs free energy 1.2 kJ/mol more negative, respectively, in EMPA compared to controls. EMPA also resulted in 26–35% higher levels of ATP and 1.7 kJ/mol more negative Gibbs free energy during reperfusion than that achieved by control hearts. EMPA also significantly improved PCr recovery by 12% over controls during the first 10 min of reperfusion (*Figure 3E*); thereafter, this difference was no longer noted. The time course of pH_i_ acidification and recovery and the level of Pi were not significantly affected by EMPA (*Figure 3F*).

### 3.6 EMPA increases ketone metabolism whilst reducing glucose utilization during ischaemia

**Figure 4:**
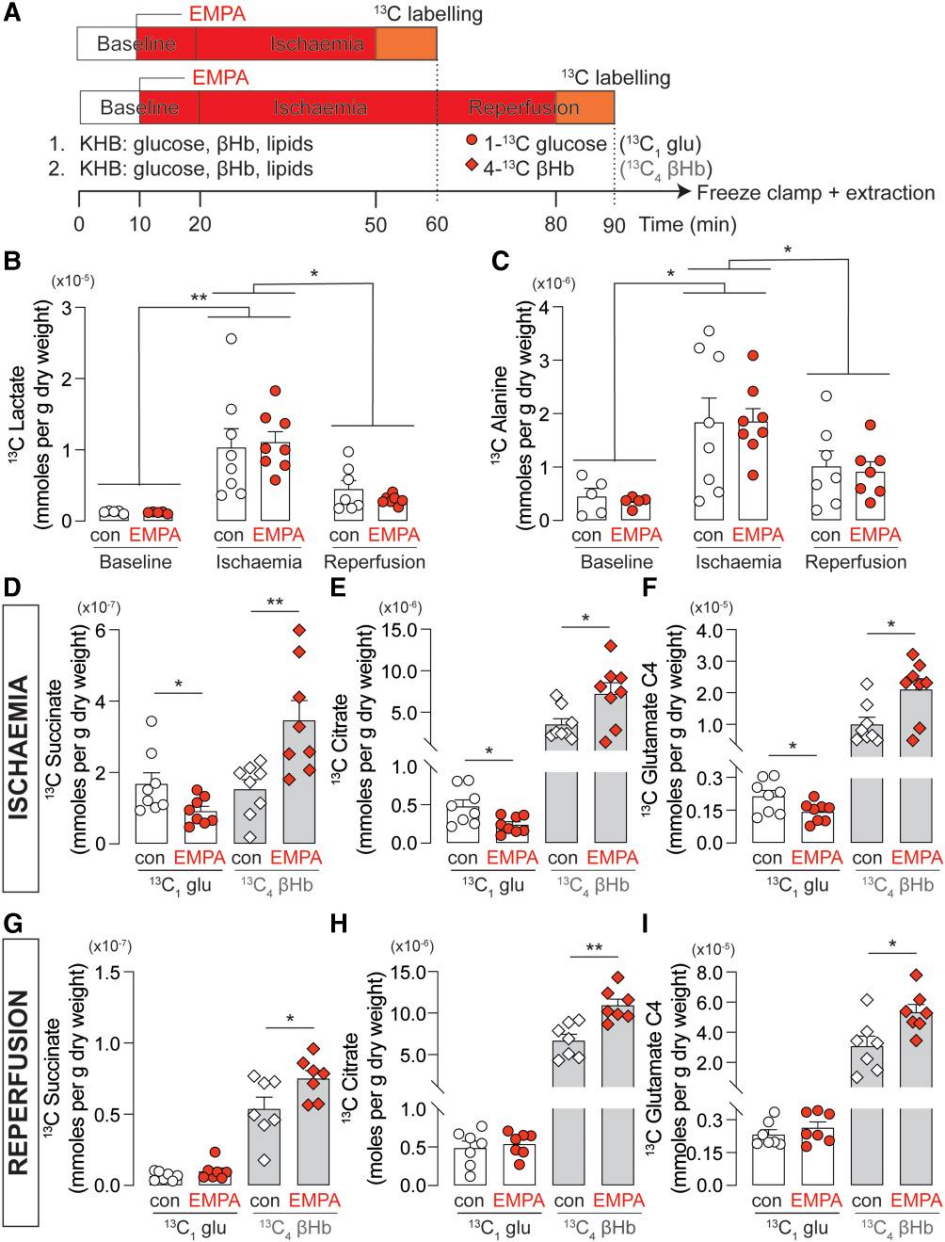
Effect of EMPA on cardiac metabolomics in ischaemia/reperfusion. (*A*) Schematic of perfusion and ^13^C labelling protocol. Hearts were perfused in ketone-supplemented KHB and perfused for 10 min to establish baseline and then treated with EMPA or DMSO for 10 min prior to 40 min low-flow ischaemia followed by 30 min of reperfusion. Hearts were ^13^C labelled for 10 min with either (i) ^13^C_1_ glucose or (ii) ^13^C_4_ βHb in the presence of EMPA or DMSO prior to snap freezing in liquid nitrogen. (*B*) ^13^C lactate and (*C*) ^13^C alanine originating from ^13^C glucose in hearts at baseline, ischaemia, and reperfusion. (*D*) ^13^C succinate (*E*) ^13^C citrate and (*F*) ^13^C glutamate originating from either ^13^C glucose or ^13^C βHb during baseline, ischaemia, and reperfusion. *n* = 8 rats per condition. *P < 0.05, and **P < 0.01. Data plotted as mean ± SEM.

Given that EMPA improved the energetics of hearts subject to I/R, we next investigated the effect of EMPA on substrate utilization in the heart. To investigate how EMPA affects mitochondrial substrate utilization under an acute pathological condition, hearts subject to low flow I/R were perfused with either ^13^C_1_ glucose or ^13^C_4_ βHb as shown in *Figure 4A*, and the extracted metabolites analysed.

As expected, ischaemia resulted in an increase in glycolytic metabolism of ^13^C_1_ glucose, with ^13^C lactate and alanine concentrations increasing by up to eight-fold and five-fold from baseline concentrations, respectively (*Figure 4B* and *C*). Reperfusion reduced the levels of these glycolytic products to levels which were not significantly different to baseline concentrations. EMPA did not affect glycolysis during baseline, ischaemia, and reperfusion, as the concentration of lactate and alanine during these periods was comparable to their respective controls.

The contribution of glucose and ketones to TCA cycle intermediates was also compared during ischaemia and reperfusion. During ischaemia, EMPA treatment resulted in a reduction of ^13^C_1_ glucose-derived succinate, citrate, and glutamate and a significant increase in ^13^C_4_ ketone-derived products (*Figure 4D–F*), indicating a decrease in glucose oxidation and a concomitant increase in ketone oxidation. After reperfusion, whereas the concentration of glucose-derived TCA intermediates was not affected by EMPA, ketone-derived intermediates remained elevated in EMPA-treated hearts when compared to the controls (*Figure 4G–I*).

## 4. Discussion

Several studies suggest that the cardioprotective effects of EMPA may be attributable to improved cardiac efficiency via enhanced cardiac ketone delivery.^4,15,24^ In this study, we present results showing a direct effect of EMPA, independent of substrate supply, on metabolic substrate preference under healthy and pathological conditions in Langendorff-perfused, non-diabetic rat hearts subject to acute low flow I/R. Specifically, EMPA treatment resulted in a switch away from glucose oxidation towards ketone oxidation during ischaemia and subsequent reperfusion. Glycolytic metabolism was not altered by EMPA treatment during baseline and ischaemia nor reperfusion. Changes in steady state ^13^C incorporation were accompanied by improved ATP and PCr levels, improved Gibbs free energy, and functional improvement during ischaemia and reperfusion with EMPA. Notably, EMPA treatment caused LV function to recover significantly better than controls from the onset of reperfusion and through the remainder of the protocol. Importantly, these alterations were observed in the absence of a change in the supply of glucose and ketone concentration in the perfusate. The findings reported in this study are of significance because it describes for the first time a direct cardiac effect that is independent of systemic ketone availability and may explain the cardioprotective benefits associated with EMPA in the heart. Until now, the effects of EMPA on cardiac ketone metabolism have been largely attributed as secondary to changes in the supply of plasma βHb through increased circulation of ketone bodies in the blood.

The ‘thrifty fuel’ hypothesis surrounding ketones in myocardial pathology reflects the fact that ketone bodies are more energetically efficient fuel than FFA (P/O 2.5 for ketones vs. 2.33 for palmitate) and more energy liberating than glucose (244 kcal/mol for ketones vs. 224 kcal/mol for glucose).^25^ When the heart is supplied with ketones, their contribution to the TCA cycle increases in a concentration dependent manner.^26^ Consistent with these previous observations, the addition of βHb in the perfusate significantly increased the concentration of ketone-derived TCA intermediates along with a substantial decrease in the contribution of glucose when compared to the metabolic profile that results from the absence of ketones (*Figure 2E and F*).

Furthermore, in the presence of βHb, the flux of ^13^C_1_ glucose through lactate and alanine was decreased significantly compared to perfusion conditions lacking this metabolic substrate (*Figure 2C* and *D*). Since these metabolites can only originate from glucose, this reduction in glycolysis is consistent with a switch in substrate preference towards ketone oxidation over glucose utilization.

### 4.1 EMPA mediates preferential cardiac ketone utilization over glucose under pathological conditions

In this study, we used ischaemia as a metabolic stressor to mimic a pathological state in an acute setting. Metabolic profiles of hearts undergoing ischaemia had upregulated glycolysis (*Figure 4B* and *C*), consistent with a metabolic phenotype expected at low oxygen conditions; reperfusion reversed these alterations, restoring the concentration of glycolytic products back to baseline levels. Whereas EMPA did not affect the glycolytic capacity of the heart in ischaemia, the drug did result in a switch in substrate preference away from glucose oxidation, towards ketone oxidation, as evidenced by the increase in the contribution of ^13^C_4_ βHb and a decrease in the contribution of ^13^C_1_ glucose in the flux of TCA intermediates generated during this period (*Figure 4E–G*).

The beneficial role of ketone metabolism is well established in the context of cardiac pathology. Previous *in vivo* data have shown that a shift towards myocardial ketone metabolism is important in maintaining adequate fuel supply for ATP oxidative production under pathological conditions. Specifically, in murine models of transverse aortic constriction, increased ketone oxidation ameliorated HF and improved cardiac contractility,^27^ whereas reduced ketone oxidation worsened the HF phenotype.^28^ In humans, ketone utilization is increased in severely failing human hearts, as part of metabolic adaptation to pathology.^29^

A recent study conducted by Santos-Gallego *et al*.^24^ showed a switch in myocardial substrate preference away from glucose and towards ketones in a pig model of M/I - in EMPA-treated animals compared to controls. Evidence for this metabolic switch was based on a cumulative observation of decrease glucose utilization, increased ketone uptake into the heart, and higher levels of the ketone metabolizing enzymes SCOT and BDH1. However, EMPA-treated animals also had increased levels of plasma ketone, thus making it difficult to distinguish between the indirect systemic effect and direct cardiac effect of the drug. Our study sheds light on this by testing the effects of EMPA under conditions of unchanging ketone supply to the heart. EMPA-treated hearts displayed a preference for ketone oxidation over glucose oxidation during the ischaemic phase, thus providing evidence that EMPA acts to directly modulate cardiac substrate preference and utilization, independent of systemically driven substrate availability.

During reperfusion, the recovery in cardiac energetics, in terms of ATP, PCr, and Gibbs free energy was significantly improved in EMPA-treated hearts compared to controls (*Figure 3C* and *D*). Higher levels of these metabolites and improvement in energetics are likely to contribute to the functional differences observed at this time (*Figure 3A*).

Interestingly, we observed that under baseline conditions EMPA reduced ketone oxidation (*Figure 2E* and *F*) but improved LV function (*Figure 3A*), without altering myocardial energetic balance (*Figure 3C* and *D*). This is in contradiction to the notion that EMPA specifically augments ketone oxidation over glucose utilization, thereby improving energetic efficiency. Although ketone bodies do release more energy compared to glucose, the latter is a more *oxygen*-efficient substrate compared to ketones (P/O 2.58 for glucose vs. 2.5 for ketones), thus suggesting that ketones are not necessarily a more ‘thrifty fuel’ than glucose under normoxic conditions. In fact, stimulating ketone oxidation rates in an aerobically perfused mouse heart did not improve cardiac efficiency nor ATP production, despite a three-fold increase in TCA cycle activity,^30^ but rather higher levels of ketone oxidation resulted in a decrease in cardiac efficiency. Thus, it is plausible that, under aerobic conditions, by reducing ketone utilization, EMPA attenuates the ketone-induced decrease in cardiac efficiency, resulting in an apparent improvement in function over controls. This observation also raises the possibility that EMPA’s mechanism of action may not be limited to solely augmenting ketone metabolism but generally modulating its utilization depending on the heart’s metabolic needs. This hypothesis requires additional experimentation which is beyond the scope of this study.

Of note, pH_i_ was not significantly affected by EMPA treatment neither at baseline and ischaemia nor reperfusion. This is of interest because an alternative proposed mechanism of action is that EMPA is a potent inhibitor of the cardiac Na^+^/H^+^-exchanger 1 (NHE-1), a Na-driven, pH-regulating membrane protein.^31,32^ Specifically, the time course of intracellular acidification was not significantly affected by EMPA during ischaemia (*Figure 3F*). This suggests that the activities of pH_i_ regulating proteins, such as NHE-1 or the sodium bicarbonate cotransporter, were not inhibited by EMPA. This result supports our previous work^23^ showing that EMPA and other related SGLT2i’s are not inhibitors of NHE-1.

Taken together, the results of this study show, for the first time, a direct cardiac effect of EMPA on myocardial substrate utilization, shifting the substrate preference towards oxidation of ketone over glucose under acute pathological conditions and constant supply of substrate. Our findings highlight the therapeutic potential of EMPA for HF and other cardiac pathologies where myocardial metabolism is affected even in non-diabetic patients and warrant further investigation.

## Supplementary material

Supplementary material is available at *Cardiovascular Research* online.

## Supplementary Material

cvad157_Supplementary_Data

## Data Availability

The data underlying this article will be shared on reasonable request to the corresponding author.

## Notes

Translational perspective Heart failure remains a huge clinical burden. Clinical trials of sodium–glucose cotransporter 2 inhibitors in patients with diabetes and heart failure have reported significant cardio-protection from empagliflozin (EMPA) treatment that appears independent of improved glycaemic control. The direct cardiac effect of EMPA in modulating ketone metabolism observed in this study raises the potential for EMPA to be used as a therapy for heart failure in both diabetic and non-diabetic patients alike.
