# Supplementary material for: Empagliflozin improves cardiac energetics during ischaemia/reperfusion by directly increasing cardiac ketone utilization
Source: Cardiovasc Res. 2023 Oct 11;119(16):2672–80. doi: 10.1093/cvr/cvad157 (PMC10730240; doi:10.1093/cvr/cvad157)
Supplement: cvad157_Supplementary_Data [file cvad157_supplementary_data.docx]

SUPPLEMENTARY MATERIAL

Empagliflozin improves cardiac energetics during ischaemia/reperfusion by directly increasing cardiac ketone utilisation

Short title: EMPA increases cardiac ketone utilisation independent of supply

Dylan CHASE^1^, Thomas R. EYKYN^1,2^, Michael J. SHATTOCK^1^, Yu Jin CHUNG^1*^

*(1) British Heart Foundation Centre of Research Excellence, King’s College London, United Kingdom*

*(2) School of Biomedical Engineering and Imaging Sciences, King’s College London, United Kingdom*

*Address for correspondence: Email: [yujin.chung@kcl.ac.uk](mailto:yujin.chung@kcl.ac.uk), The Rayne Institute, 4^th^ Floor, Lambeth Wing, St Thomas’ Hospital, London SE1 7EH, UK. Telephone: +44 (0)20 7188 0945

Manuscript category: Original Article


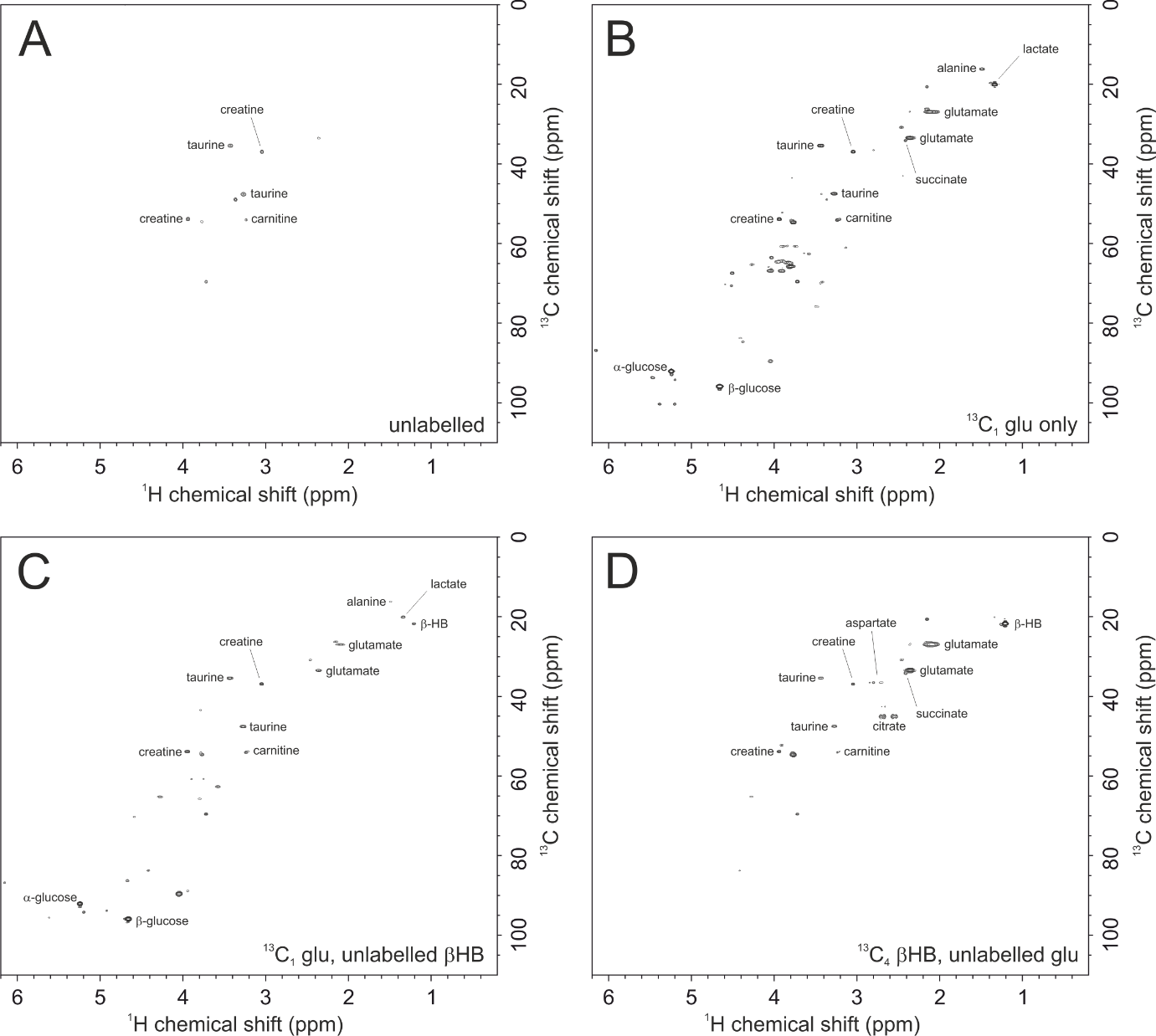


**Figure S1.** Gradient selected ^1^H/^13^C HSQC experiments acquired on a Bruker Avance III 400 MHz Spectrometer 9.4T vertical-bore magnet equipped with a BBO probe under the various labelling condition reported in the main text. A) Unlabelled heart showing natural abundance ^1^H/^13^C peaks from only the most concentration metabolites. B) Heart labelled with ^13^C_1_ glucose in the absence of βHB. C) Heart labelled with ^13^C_1_ glucose in the presence of unlabelled βHB. D) Heart labelled with ^13^C_4_ βHB in the presence of unlabelled glucose.
